# A promising nucleic acid therapy drug: DNAzymes and its delivery system

**DOI:** 10.3389/fmolb.2023.1270101

**Published:** 2023-09-11

**Authors:** Lang Xiao, Yan Zhao, Meng Yang, Guangxin Luan, Ting Du, Shanshan Deng, Xu Jia

**Affiliations:** ^1^ School of Basic Medical Sciences, Chengdu Medical College, Chengdu, Sichuan, China; ^2^ Sichuan Key Laboratory of Noncoding RNA and Drugs, Chengdu Medical College, Chengdu, Sichuan, China; ^3^ Life Science and Engineering, Southwest Jiaotong University, Chengdu, Sichuan, China

**Keywords:** DNAzymes, *in vitro* selection, catalytic function, RNA cleavage, therapeutic agent, gene expression

## Abstract

Based on the development of nucleic acid therapeutic drugs, DNAzymes obtained through *in vitro* selection technology in 1994 are gradually being sought. DNAzymes are single-stranded DNA molecules with catalytic function, which specifically cleave RNA under the action of metal ions. Various *in vivo* and *in vitro* models have recently demonstrated that DNAzymes can target related genes in cancer, cardiovascular disease, bacterial and viral infection, and central nervous system disease. Compared with other nucleic acid therapy drugs, DNAzymes have gained more attention due to their excellent cutting efficiency, high stability, and low cost. Here, We first briefly reviewed the development and characteristics of DNAzymes, then discussed disease-targeting inhibition model of DNAzymes, hoping to provide new insights and ways for disease treatment. Finally, DNAzymes were still subject to some restrictions in practical applications, including low cell uptake efficiency, nuclease degradation and interference from other biological matrices. We discussed the latest delivery strategy of DNAzymes, among which lipid nanoparticles have recently received widespread attention due to the successful delivery of the COVID-19 mRNA vaccine, which provides the possibility for the subsequent clinical application of DNAzymes. In addition, the future development of DNAzymes was prospected.

## 1 Introduction

In recent years, nucleic acids have been used as gene regulatory tools or therapeutic agents for treating diseases due to their molecular recognition ability, programmability ease of synthesis, and chemical modification (Wang et al., 2022). Compared to traditional therapies, nucleic acid therapy fundamentally treats diseases through gene suppression, addition, replacement, or editing. With more and more nucleic acid therapies being approved, nucleic acid-based therapy has become an independent field of treatment (Sridharan and Gogtay, 2016; Kulkarni et al., 2021). The first nucleic acid drug used to treat cytomegalovirus retinitis in immunocompromised patients has been approved by the US Food and Drug Administration (Rosenberg et al., 2000). In the following decade, many genes were targeted by specialized engineering reagents from different categories of nucleic acid drugs to experimental disease models, sparking a wave of gene silencing strategies (Bhindi et al., 2007). Nucleic acid therapy drugs include siRNA, an antisense oligonucleotide (ASO), ribozyme, aptamer, and DNAzymes.

In contrast, DNAzymes attract more attention in nucleic acid therapy because of their small molecular weight, higher stability, excellent programmability, and low cost. Unlike ribozymes, natural DNAzymes have not yet been found in nature. All existing DNAzymes are obtained from libraries containing 10^15^ DNA sequences through *in vitro* selection technology. The first DNAzyme was reported by Breaker and Joyce to be used for RNA cleavage in 1994, and they found that this DNAzyme has great potential in downregulating mRNA expression (Breaker and Joyce, 1994). Since then, the application of DNAzymes in biomedicine has been everywhere. Here, we focus on applying DNAzymes in treating diseases, including cancer, cardiovascular diseases, bacterial and viral infections, and central nervous system disease. However, DNAzymes are still subject to degradation by nuclease in practical applications, which has limitations such as low stability and insufficient cell uptake. Therefore, we should critically analyze the application of DNAzymes in biomedicine to improve the effectiveness and prospect of DNAzymes in treatment. Finally, given these limitations, we discuss some promising delivery systems to improve the safety and effectiveness of DNAzymes for further clinical application.

## 2 Development and characteristics of DNAzymes

**FIGURE 1:**
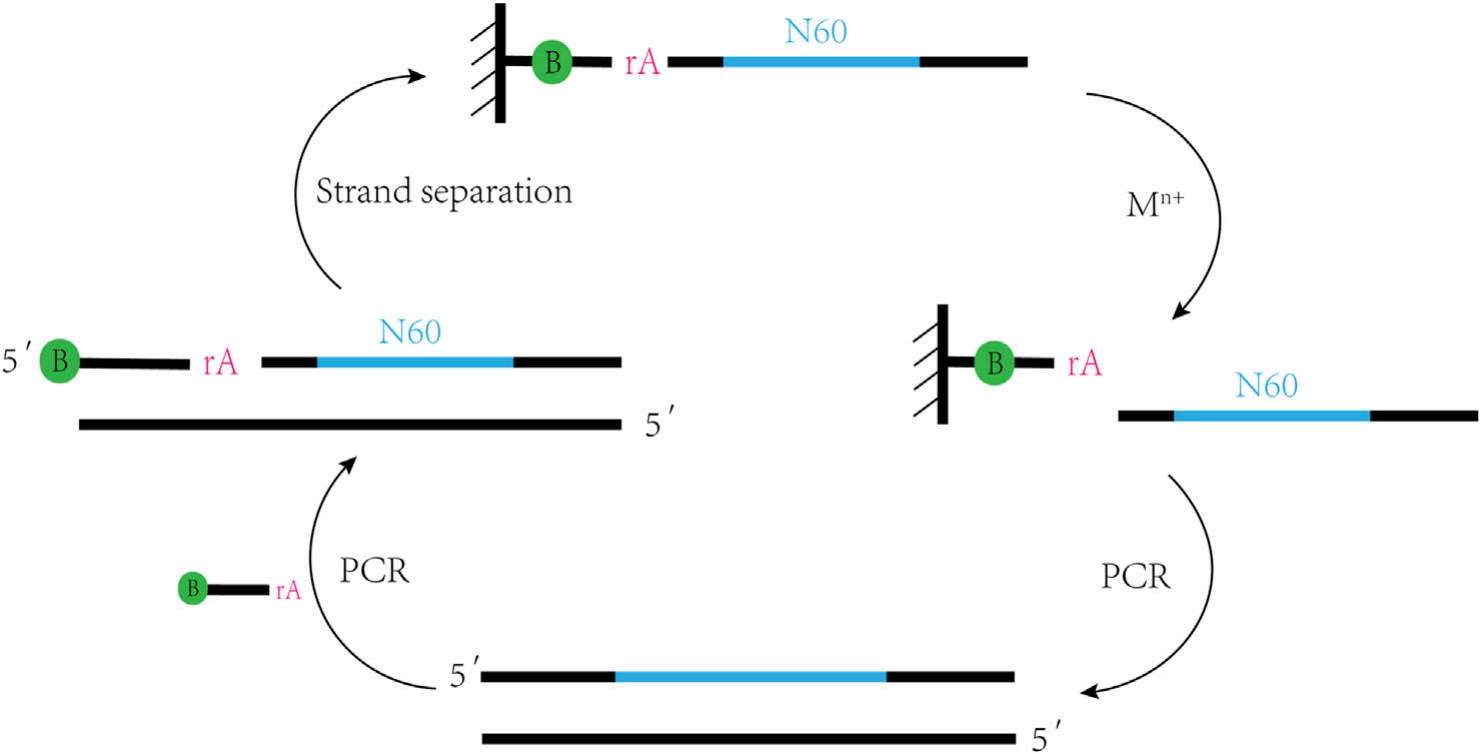
*In vitro* selection strategy of DNAzymes. This selection strategy involves repeated PCR rounds to incorporate biotin streptavidin chromatography to separate single-stranded nucleic acids and M^n+^ catalyzed RNA cleavage; PCR regenerates the pool to enrich DNAzymes that can cleave RNA.

DNAzymes are single-stranded DNA molecules with catalytic function, obtained by Breaker and Joyce through *in vitro* selection technology in 1994 (Breaker and Joyce, 1994). Firstly, we will briefly review the strategy for *in vitro* selection of DNAzymes (Figure 1). In an initial selection DNA sequence library, consisting of a 60-nucleotide random region and two constant regions on both sides for primer binding, ribo-adenine (rA) is used as a specific cleavage site and biotin is labeled at the 5´ end of the DNA to fix the library on a streptavidin column. Under the action of metal ions, the library with active structure is cleaved at the rA site and released from the chromatographic column. The cleavage products are amplified by PCR to regenerate the library and enrich DNAzymes that can cleave RNA. Overall, the technology steps are simple and does not require expensive costs, making DNAzymes easy to synthesize. With the advancement of DNA synthesis technology, fluorescent group labeling can be well attached to libraries and be incorporated with fluorescent groups and quenching agents next to the cleavage site to achieve synchronization of cleavage reactions and fluorescence signals (Mei et al., 2003).

**FIGURE 2:**
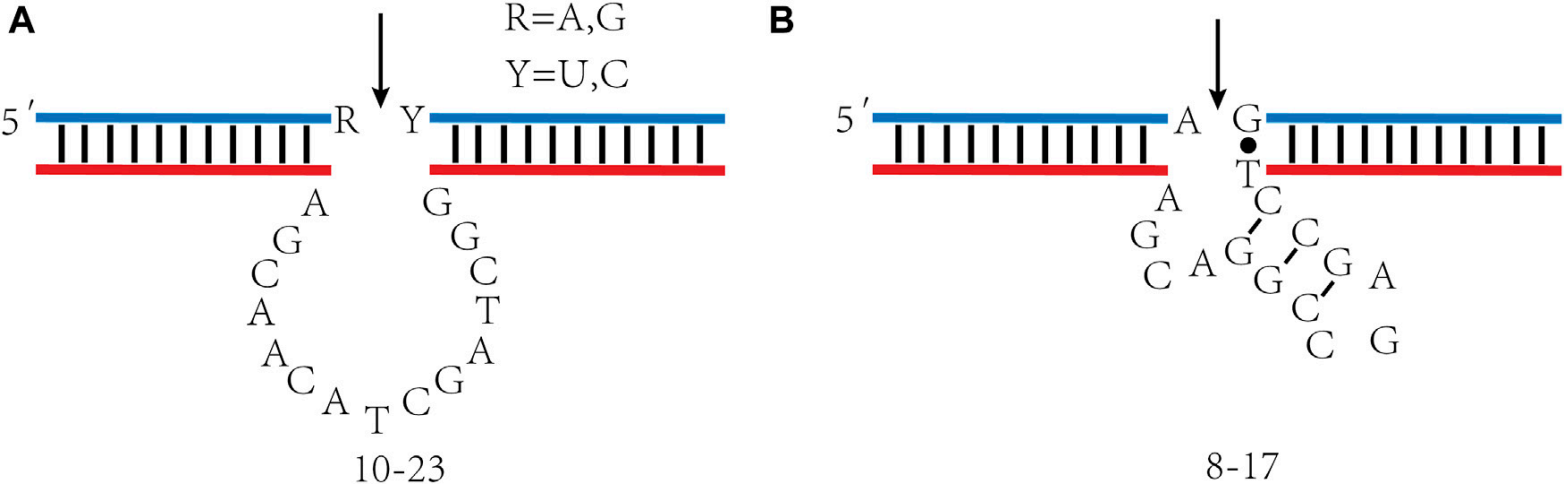
10–23 and 8–17 DNAzyme´s secondary structure. **(A)** 10–23 DNAzyme; **(B)** 8–17 DNAzyme. The arrowheads denote cleavage site.

DNAzymes comprise two domains, one catalytic domain, and two substrate-binding domains. It combines with RNA substrate through Watson-Crick base pairing, catalyzes specific cleavage of RNA under the action of metal ions, and forms 2´, 3´—cyclic phosphate and 5´—hydroxy terminal. In addition to catalyzing RNA cleavage, DNAzymes also catalyze a series of chemical reactions, including DNA binding (Cuenoud and Szostak, 1995), DNA cleavage (Carmi et al., 1998), RNA binding (Purtha et al., 2005), and DNA phosphorylation (Camden et al., 2016). The research and application of RNA cleavage DNAzymes are the most extensive (Silverman, 2005). The 10–23 and 8–17 DNAzyme obtained by Santoro et al., in 1997 are RNA-splitting DNAzymes studied extensively. These two DNAzymes can cut almost any RNA substrate under simulated physiological conditions (Santoro and Joyce, 1997). 10–23 DNAzyme comprise 15 deoxynucleotides, constituting a catalytic domain and two substrate recognition domains. Each domain has 7–8 deoxynucleotides (Figure 2A). This enzyme cleaves at the phosphodiester between unpaired purines and paired pyrimidine residues, targeting different RNA substrates by altering the sequence of substrate recognition domains. A significant advantage is that 10–23 DNAzyme cleave all purine pyrimidine connections, which provides excellent flexibility for targeting specific sites of RNA sequences. The 8–17 DNAzyme´s catalytic domain consists of 13 nt, including a short inner stem ring and an unpaired region of 4–5 nt (Figure 2B). This loop has a fixed sequence of 5´—AGC—3´ and extending the stem or changing the order of the loop will not demonstrate catalytic activity. The unpaired region connecting the 3´ half of the stem to the downstream substrate binding domain has sequence 5´—WCGR—3´ or 5´—WCGAA—3´ (W = A or T; R = A or G), the variant with sequence 5´—TCGAA—3´ in this region exhibits the highest level of catalytic activity.

At present, these two DNAzymes have been widely studied and discussed. 10–23 DNAzyme have been reported as a useful therapeutic agents by many studies due to their excellent cutting efficiency. In contrast, 8–17 DNAzyme is relatively few. Regarding structure and catalytic mechanism, the lack of detailed information on spatial arrangement and metal coordination sites has hindered our in-depth understanding of DNAzymes catalysis. So far, we have only obtained the crystal structure of RNA-linked DNAzyme 9DB1 and 8–17 DNAzyme (Ponce-Salvatierra et al., 2016; Liu et al., 2017a). According to this available crystal structure, 8–17 DNAzyme, like other ribozymes, catalyzes RNA cleavage through a general acid-base mechanism, with a specific G residue (G14) acting as a general base and metal coordinated water molecules acting as a general acid (Cepeda-Plaza and Peracchi, 2020). However, no conclusions have been drawn regarding the structural information of 10–23 DNAzyme catalyzed RNA cleavage.

The application of DNAzymes mainly involves two fields, one as a nucleic acid therapy drug and the other for biosensors. Firstly, some excellent reviews published in recent years have discussed in detail the application of DNAzymes in biosensing, many DNAzymes are designed as various biosensors to detect relevant targets in organisms (McConnell et al., 2021; Ma and Liu, 2020; Liu et al., 2017b). In the field of medical treatment, DNAzymes have been widely studied from the moment they are obtained. The 10–23 DNAzyme obtained in 1997 sparked a wave in the following time and related reviews published on this topic were also endless (Silverman, 2005; Khachigian, 2002; Patil et al., 2005). A recent review provides a detailed overview of the structural design and clinical application of DNAzymes, they deeply discussed strategies for improving the catalytic activity and stability of DNAzymes in structural mechanism, providing a good novelty (Yan et al., 2023). As it said, DNAzymes enter a rapid development stage in the early stages, but its clinical development has been relatively slow in recent years, indicating that DNAzymes still have a long way to go compared to other nucleic acid therapy drugs. However, considering that DNAzymes are not easy to miss the target *in vivo* and have low cytotoxicity and low immunogenicity, leading to DNAzymes becoming the focus of many researchers. This article mainly discussed using DNAzymes as a drug *in vivo* and *in vitro* to inhibit the expression of disease-related target genes in the past 20 years, hoping to provide a good direction for the development of DNAzymes in the field of treatment and diagnosis in the future.

## 3 Application of DNAzymes in disease treatment

Since the acquisition of DNAzymes, their greatest feature is to achieve specific RNA cleavage and to target different RNA substrates by changing the base sequence in the catalytic ring, which has become a useful tool for many researchers to treat diseases. It has been proved that DNAzymes can target the expression of related target genes in cancer, cardiovascular disease, bacterial and viral infection and central nervous system disease. Next, we will focus on DNAzymes targeting these disease targets to block the occurrence of diseases.

### 3.1 DNAzymes targeted therapy for cancer

#### 3.1.1 Inhibition of oncogene expression

The first cancer treatment with DNAzymes was reported by Wu et al. (1999). They designed three DNAzymes and found that these DNAzymes could effectively cut two oncogenes, p210 bcr-abl, and p190 variants, *in vitro*. In target K562 cells expressing p210 bcr-abl, DNAzymes specifically inhibited the expression of p210 bcr-abl protein in K562 cells by about 40% and inhibited cell growth by more than 50%. In addition, they also measured the activity of DNAzymes in freshly isolated CD34^+^ bone marrow cells from CML patients and found that DNAzymes could specifically inhibit the growth of bcr abl positive CFU mixed colonies, reaching 53%–80%. Since then, more and more studies have shown that DNAzymes can inhibit cancer development at different stages by targeting different oncogenes, tumor formation-related factors, or other cancer targets.

#### 3.1.2 Inhibition of tumor-related factor expression

β1 Integrins have been shown to have higher expression levels in the metastasis of various cancers and play an important role through extracellular matrix remodeling (Rathinam and Alahari, 2010; Masumoto et al., 1999; Arao et al., 2000). Wiktorska et al. (Niewiarowska et al., 2009) designed a specific DNAzyme (DEβ1) targeting the human β1 integrin subunit, Which significantly inhibited the expression of Integrin subunits in endothelial cells and K1 cells at the level of mRNA and protein synthesis and effectively eliminated the capillary formation of microvascular endothelial cells in fibrin and Matrigel. Subsequently, they conducted *in vivo* experiments in mice and found that DEβ1 continuation of 1 week significantly reduced the tumor size and microvascular count produced by prostate cancer cells (PC-3) and colon adenocarcinoma cells (CX1.1) (Niewiarowska et al., 2009). In addition, DEβ1 was also measured in various cancer cell lines (CX1.1, HT29, LOVO, LS180, PC-3), Which effectively inhibited the expression of β1 integrin mRNA and eliminated the invasiveness of CX1.1, HT29, LS180, LOVO and PC-3 cells *in vitro* (Wiktorska et al., 2010). A study also tested the inhibitory efficiency of DEβ1 and siRNA on β1 integrins mRNA, and they could effectively inhibit the growth of PC3 and HT29 colon cancer cells in the mouse xenotransplantation model. In contrast, the inhibition effect of DNAzymes *in vitro* was not as good as that of siRNA, but it showed higher efficiency in blocking tumor growth *in vivo* (Wiktorska et al., 2013).

Insulin and insulin-like growth factor (IGF) are key regulatory factors for energy metabolism and growth. Studies have shown that these regulatory factors are important in tumor formation (Pollak, 2008). Among them, the reactivation and imbalance of IGF-IIP3 are related to weakened cell apoptosis and proliferation of many liver cancer cell types (Zhang et al., 2013). Zhang et al. (2013) designed specific DNAzymes (DRz1 and DRz2) targeting IGF-IIP3 mRNA and transfected DRzs into hepatoma cells. They found that DRz1 inhibited the expression of IGF-IIP3 by nearly 50%, significantly inhibited cell proliferation and induced cell apoptosis, and the expression of IGF-IIP3 in SMMC-7721 cells 24 h after transfection was related to the increase of caspase - 9 activities. In all experiments, the activity of DRz2 is second only to DRz1.

Similarly, DNAzymes also target other related-cancer targets (Table 1), which will not be discussed further here. From the current test results, DNAzymes effectively inhibit tumor-related factors´ expression *in vivo* and *in vitro*, thereby blocking the growth and migration of tumors, angiogenesis, inducing tumor cell apoptosis, and enhancing tumor radiosensitivity. It is worth noting that Dz13 is a DNAzyme that cleaves c-Jun mRNA. The basic region-leucine zipper protein (c-Jun) is related to cell proliferation, apoptosis, and angiogenesis. It has been found that Dz13 can inhibit tumor-induced angiogenesis, new intima formation after arterial injury and control inflammatory reactions (Elahy and Dass, 2011). Other studies showed that Dz13 could inhibit more types of tumors, including skin, breast, prostate, osteosarcoma, and liposarcoma, which have been proved in experimental animal model tumors (Dass et al., 2010; Cai et al., 2012). Based on these studies, we think that it will be possible for DNAzymes to become a potential cancer therapeutic agent.

**TABLE 1:** DNAzymes target cancer-related genes.

mRNA target	DNAzyme	Result	References
Caspase-2	Dz13	Induction of cell apoptosis and inhibition of tumor growth	Dass et al. (2010)
Caspase-10	Dz13	Induction of cell apoptosis and inhibition of tumor growth	Dass et al. (2008b)
Survivin	SD	Induction of cell apoptosis	Liang et al. (2005)
bcl-xL	DT 882	Inhibition of tumor growth and chemosensitization	Yu et al. (2014)
uPAR	Dz372, Dz483, and Dz720	Inhibition of cell invasion and proliferation	de Bock et al. (2005)
MMP-9	MMP-9 DNAzymes	Inhibition of cell invasion, adhesion, and proliferation	Yang et al. (2009a)
Egr-1	DzF、ED5	Inhibition of tumor growth and angiogenesis	Fahmy et al. (2003)
VEGFR-1	DT18	Inhibition of tumor growth and change of Vascular permeability	Shen et al. (2013)
VEGFR-2	VEGFR-2 Dz	Inhibition of tumor growth and reduction of vascular density	Zhang et al. (2002)
C-jun	Dz13	Induction of apoptosis	Zhang et al. (2004)
LMP1	Dz1, Dz7, and Dz10	Inhibition of cell proliferation, induction of apoptosis, and radiosensitization	Lu et al. (2008)
K-Ras	DZ-A	Sensitization to chemo- and radiation therapies	Yu et al. (2009)
EGFR	Ex19delDZ	Reduction in cell viability, suppression of cell proliferation, and induction of apoptosis	Jang et al. (2018)
Aurora kinase A	DZ2	Suppression of cell growth, inhibition of cell cycle progression, induction of cell apoptosis, and attenuation of cell migration	Qu et al. (2008)
Akt1	Dz2	Inhibition of cell proliferation and induction of apoptosis	Yang et al. (2009b)
PKCalpha	DRz4	Inhibition of cell proliferation and induction of apoptosis	Sioud and Leirdal (2000), Sorensen et al. (2002)
protein kinase Raf-1	Raf-1 Dz	Inhibition of cell growth and proliferation	Iversen et al. (2002)
PML/RARalpha	DZ1 and DZ3	Inhibition of cell proliferation, reduction of cell viability, and induction of apoptosis	Kabuli et al. (2004)

### 3.2 DNAzymes targeted therapy for cardiovascular diseases

#### 3.2.1 Inhibition of Egr-1

Early growth factor (Egr-1) is a zinc finger transcription factor that is known to upregulate vascular smooth muscle cells (SMC) and endothelial cells through mechanical injury under various conditions (Silverman et al., 1999). This function is determined by the experiment of antisense oligonucleotide targeting the Egr-1 start codon or the sequence encoding one of the key zinc fingers required for Egr-1 DNA binding. Antisense oligonucleotide inhibits the induced synthesis of Egr-1 protein in a sequence-specific manner and inhibits DNA synthesis and cell replication (Santiago et al., 1999a). Therefore, Santiago et al. designed a specific DNAzyme (ED5) targeting Egr-1 mRNA, which proved that ED5 effectively inhibited Egr-1 mRNA and protein synthesis in SMC nuclei under fluorescein labeling (Santiago et al., 1999b). In addition, it was found that ED5 effectively inhibited Egr-1 mRNA induction and neointimal formation after balloon injury of the carotid artery wall in rats (Santiago et al., 1999b). It is worth noting that this study is the first to prove that DNAzymes have therapeutic effects *in vivo.*

In addition, Egr-1 also plays an important role in response to ischemia-reperfusion injury in other cell types and organ systems. Bhindi et al. (2006) delivered DNAzymes locally to the rat myocardial cell model *in vitro*. They found that ED5 could selectively inhibit the upregulation of Egr-1 mRNA and protein after myocardial ischemia-reperfusion injury in rats. Neutrophil infiltration, intercellular adhesion molecule mRNA and protein expression and myocardial infarction size were reduced in the treated animal myocardium confirming that Egr-1 is a key factor in myocardial ischemia-reperfusion injury, providing support for further targeting Egr-1 for the treatment of ischemia-reperfusion injury.

#### 3.2.2 Inhibition of plasminogen activator inhibitor (PAI)-1

Another important marker of cardiovascular disease is PAI-1, which plays an important role in inducing the formation of new blood vessels in infarcted myocardium. Xiang et al. (2004) injected DNAzymes targeting PAI-1 mRNA into the infarct surrounding area of rats with AMI and found that the expression of PAI-1 decreased within 2 weeks after treatment. By increasing the migration of labeled human adult bone marrow-derived angioblasts and the density of human-derived capillaries, it proved that the neovascularization of infarct tissue was improved, indicating that the inhibition of PAI-1 on the production of plasmin and the adhesion medium had been offset by DNAzymes treatment. Further studies revealed that the delivery of DNAzymes targeting PAI-1 into the heart of infarcted mice inhibited the expression of PAI-1 and improved neovascularization, and reduced apoptosis in the tissues around the infarction (Xiang et al., 2005a).

#### 3.2.3 Inhibition of vitamin D upregulated protein 1

DNAzymes are also used to study the overexpression of vitamin D3 upregulated protein 1 (VDUP1) caused by myocardial ischemia and oxidative stress. VDUP1 is a key mediator of oxidative stress on various cell processes through its downstream effects on apoptosis signal kinase 1 (ASK1) and p38 mitogen-activated protein kinase (MAPK). Xiang et al. (Xiang et al., 2005b) found that under H_2_O_2_ stress conditions, transfection of H9C2 cardiomyocytes with a sequence-specific VDUP1 DNAzymes significantly downregulated the expression of VDUP1 mRNA, reduced cell apoptosis and enhanced cell survival. Direct intracardiac injection of DNAzymes in acute myocardial infarction also significantly decreased the expression of myocardial VDUP1 mRNA and led to a long-term reduction of ASK1 activity and apoptosis. In addition, it effectively reduced the formation of myocardial scars. These results indicate that VDUP1 plays a direct role in the process of myocardial ischemia and oxidative stress and also confirm that DNAzymes down-regulating the expression of VDUP1 mRNA help to improve cardiac function and prevent left ventricular remodeling.

#### 3.2.4 Inhibition of c-jun

The role of c-jun in tumor disease has been revealed using Dz13, and this transcription factor also plays a regulatory role in cardiovascular disease. Khachigian et al. (2002) confirmed that poor c-Jun expression was induced after arterial injury, Dz13 targeting c-jun mRNA significantly inhibited c-Jun expression induced by vascular smooth muscle cells and blocked vascular smooth muscle cell proliferation, also demonstrated in carotid arteries of rats with *in vitro* scratch injury and *in vivo* injury. Subsequent studies also further demonstrated the direct role of c-jun in various angiogenesis models, Dz13 may be a potential therapeutic agent for anti-tumor and cardiovascular diseases (Zhang et al., 2004).

### 3.3 DNAzymes targeted therapy for inflammatory diseases

#### 3.3.1 Inhibition of TGF- β

In glomerulonephritis, it usually leads to the proliferation of mesangial cells and the accumulation of extracellular matrix (ECM). Due to the important role of transforming growth factors-β (TGF-β) in regulating cell proliferation, differentiation and immune response, especially in regulating ECM accumulation, inhibiting the expression of TGF-β is an effective strategy for treating glomerular diseases. Isaka et al. (2004) designed a specific DNAzyme (TGFDE) targeting TGF-β1 mRNA. In cultured rat mesangial cells, TGFDE effectively inhibited TGF-β1 mRNA expression, thereby blocking the expression of type I collagen. Three days after disease induction, they introduced TGFDE or scrambled DNAzymes (TGFSCR) into the anti-Thy-1 model of nephrotic rats through electroporation and found that TGFDE effectively inhibited the expression of TGF-β1, smooth muscle actin and type I collagen in nephrotic rats on the seventh day compared with untreated and TGFSCR-transfected nephrotic rats.

#### 3.3.2 Inhibition of INOS

Studies have shown that lipopolysaccharide (LPS) induced acute inflammatory response can increase iNOS. INOS is a key enzyme in synthesizing nitric oxide in organisms, and blocking its production can become a potential therapeutic strategy for treating LPS-induced inflammatory reactions. Verma et al. (2010) reported a specific DNAzyme targeting iNOS mRNA. In early studies, they proved that this DNAzyme effectively inhibited the expression of iNOS mRNA in LPS-stimulated J774 mouse macrophages. Later, they injected two DNAzymes into the BALB/c mouse model of LPS-induced fatal systemic inflammation. The results showed that the infiltration and edema of white blood cells in the mice treated with DNAzymes were significantly reduced, and the contents of IL-12, IL-1, TNF -α, and IFN-γ in serum were also significantly reduced. Therefore, this iNOS-specific DNAzyme has a good role in treating of systemic inflammatory reactions.

#### 3.3.3 Inhibition of GATA-3

GATA-3 is an important transcription regulatory factor in chronic inflammatory diseases, responsible for the production of key inflammatory cytokines IL-4, IL-5, IL-9, and IL-13 by Th2-lymphocytes and innate lymphoid cells 2 (ILC2). Downregulating GATA-3 leads to a reduced expression of these cytokines. Therefore, GATA-3 has become an ideal target for treating chronic inflammatory diseases. For example, allergic bronchial asthma is a chronic airway inflammatory disease. Sel et al. (2008) selected the most active DNAzyme (gd21) targeting GATA-3 mRNA. In the allergic mouse model, they confirmed that intranasal administration of gd21 effectively inhibited airway inflammation and mucus production, and blocked the development of acute allergic airway inflammation’s high response to acetylcholine. It was worth noting that gd21 was as effective as other antisense molecules and did not exhibit significant off-target effects. Subsequently, the biological distribution and toxicity characteristics of Dz gd21, named hgd40, after inhalation exposure in mice, rats, and dogs further confirmed that this formulation could effectively distribute to the lesion site without causing serious adverse events (Turowska et al., 2013; Fuhst et al., 2013). Hgd40 has been studied and evaluated in clinical trials of allergen-induced asthma response, TH2-driven-asthma patients, and chronic obstructive pulmonary disease patients (COPD). From the current experimental results, hgd40 is safe and effective, and effectively weakens the inflammatory response regulated by Th2 lymphocytes and the production of sputum eosinophils (Krug et al., 2015; Homburg et al., 2015; Greulich et al., 2018).

In addition, hgd40 is also undergoing clinical treatment for atopic dermatitis and ulcerative colitis, and the results show that this preparation targets cleaving GATA-3 mRNA and has achieved good therapeutic effects. Recently, a study in mice administered hgd40 via rectal administration showed that compared to the control group, the colon tissue of mice treated with hgd40 significantly reduced the expression of GATA-3 mRNA, the content of some inflammatory factors IL6, IL9, and IL13 also significantly decreased (Popp et al., 2017). These studies indicate that hgd40 is a potential candidate for treating inflammatory diseases. So far, Sterna Biologicals is the only company that makes GATA-3 a proprietary drug, the company is developing an oral formulation of hgd40 for phase IIb clinical development in patients with moderate to severe ulcerative colitis, and the initial feasibility work has been completed. If the later clinical trials are successful, commercialization will ultimately be achieved.

### 3.4 DNAzymes targeted therapy of drug-resistant bacterial infections

#### 3.4.1 Inhibition of drug resistance gene expression

Many studies have shown that DNAzymes target the mRNA in antibiotic resistance genes to restore the sensitivity of drug-resistant bacteria to antibiotics. *Staphylococcus aureus* β—Lactam resistance is mediated by the mecA gene, which encodes low-affinity penicillin-binding protein 2a (PBP2a), the expression of PBP2a is regulated by two upstream genes, mecR1 and mecl (Peacock and Paterson, 2015). In addition, over 90% of *Staphylococcus aureus* also produces blaZ gene encoded β—Lactam, which makes it resistant and contains blaZ regulatory sequences (blaI and blaR1) similar to mecA regulatory factors in sequence and function (Hackbarth and Chambers, 1993). BlaI and blaR1 not only regulate the blaZ gene, but also participate in regulating mecA (Hackbarth and Chambers, 1993). Studies have confirmed that mecA is co-regulated by blaI and mecl in clinical isolates of *Staphylococcus aureus* (Lewis and Dyke, 2000; McKinney et al., 2001). Therefore, these regulator genes have become attractive targets for the action of DNAzymes. In a series of experiments conducted by Hou et al., they first designed thiophosphate DNAzymes that separately targeted blaR1 and mecR1 mRNA, named PS-DRz602 and PS-DRz147, After PS-DRz602 and PS-DRz147 were introduced into the *Staphylococcus aureus* strain WHO-2, they found that these two specific DNAzymes inhibited the expression of mecA and blaZ in a concentration-dependent manner, and restored the drug resistance sensitivity of *staphylococcus aureus* WHO-2 to oxacillin (Hou et al., 2007a; Hou et al., 2007b). Later, they used two thiophosphate DNAzymes targeting blaR1 and mecR1 respectively. The results showed that they could increase the sensitivity of all *Staphylococcus aureus* strains (Hou et al., 2011).

#### 3.4.2 Inhibition of growth and metabolic pathways

In addition to inhibiting the expression of drug-resistant genes, DNAzymes also block the biosynthesis and metabolic pathways of some drug-resistant bacteria, thereby inhibiting the growth and reproduction of bacteria. For example, the ftsZ gene plays an important role in bacterial cell division. Tan et al. (2004) constructed an expression vector regulated by tetracycline, which generated DNAzymes specifically cleaving ftsZ mRNA in bacterial cells. The generated DNAzymes could effectively inhibit the expression of the ftsZ gene and bacterial cell proliferation. Similarly, the survival of *Mycobacterium tuberculosis* in macrophages depends on tryptophan-aspartate-containing coat protein (TACO). Using this vector expressing DNAzymes could also effectively reduce the expression of TACO, thereby inhibiting the survival of *M. tuberculosis* (Li et al., 2010).

In addition, the “probable ATP-binding component of ATP transporter” plays an important role in the resistance of *Pseudomonas aeruginosa* to fluoroquinolones (Zhou et al., 2006). Zhou et al. (2006) designed a specific DNAzyme targeting the mRNA of the “probable ATP-binding component of ATP transporter” in *Pseudomonas aeruginosa*. The results showed that DNAzymes successfully reduced the resistance of *Pseudomonas aeruginosa* to ciprofloxacin by inhibiting the expression of the mRNA of the gene, and the inhibitory expression of the gene was positively correlated with the accumulation of ciprofloxacin in the strain, proving the role of the protein in active drug efflux.

However, the study of DNAzymes in treating drug-resistant bacterial infections *in vivo* has yet to be reported. Therefore, it is necessary to explore further the application of DNAzymes in the field of bacterial resistance and strive to see DNAzymes as an antibacterial agent put into clinical application as soon as possible.

### 3.5 DNAzymes targeted therapy for virus infection

#### 3.5.1 Inhibition of CCR5 and CXCR-4

CCR5 is a G protein-coupled receptor, which not only regulates the transport and effector functions of memory/effector T lymphocytes, macrophages, and immature dendritic cells but also serves as the main Coreceptor of the human immunodeficiency virus (HIV-1) (Oppermann, 2004). To prevent HIV-1 from entering and fusing into cells, Goila et al. (Goila and Banerjea, 1998) designed a specific DNAzyme targeting CCR5 mRNA and found that the DNAzyme effectively cut the full-length CCR5 gene transcript *in vitro* and effectively reduced CCR5 mediated cell membrane fusion when introduced into mammalian cells. Later, they designed a specific-DNAzyme targeting another important factor receptor (CXCR-4). When these two DNAzymes were placed in tandem, they showed higher sequence-specific cleavage activity than CCR5 DNAzyme (Basu et al., 2000). Therefore, these DNAzymes further block the infection and transmission of human immunodeficiency virus by specifically interfering with the function of the HIV-1 receptor, but the practical application *in vivo* still needs further research and evaluation.

#### 3.5.2 Inhibition of TAT and TAR

Some researchers also designed some DNAzymes to target the regulatory proteins TAT and REV, which play an important role in HIV-1 transcription and replication. These DNAzymes target the prediction loop of TAT or TAT/REV mRNA. The results showed that the cutting effect of a single DNAzyme 5,944 targeting only the TAT region was not good, but the DNAzyme 5,970 targeting the overlap of TAT and REV had strong cutting activity. When the two DNAzymes were placed in tandem, Only DNAzyme 5,970 maintained the activity of specifically cutting target RNA under simulated physiological conditions (Unwalla and Banerjea, 2001a). In addition, the DNAyme 5,970 with poly-G bundle at the 3´ end was directly absorbed by human macrophages and inhibited HIV-1 gene expression in the transient expression system (Unwalla and Banerjea, 2001b). Another unique stem ring structure called TAR exists at the 5´ end of all HIV-1 transcripts, which binds to TAT and other cellular proteins to play a transcriptional control role. Chakraborti et al. (Chakraborti and Banerjea, 2003) screened several DNAzymes containing 10–23 catalytic motifs and a single DNAzyme containing 8–17 catalytic motifs to target HIV-1 TAR mRNA. They found that DNAzyme 475 with catalytic motif could show moderate cleavage activity without Mg^2+^ and showed obvious virus resistance in transfected T lymphocytes, human peripheral blood mononuclear cells, or chronic infection cell lines.

In addition to the above targets, DNAzymes also targeted other important HIV-1 targets, including the most conservative P24 Gag and Nef regions (Sriram and Banerjea, 2000; Dash and Banerjea, 2004; Sood et al., 2007), VprB and C proteins that mediate the G2 cell cycle (Bano et al., 2007), the accessible regions of the dimer start site (DIS) and primer binding site (PBS) of 5´—UTR (Jakobsen et al., 2007). For these targets, DNAzymes showed inhibition of HIV-1-specific gene expression. Many studies on DNAzymes targeting the expression of genes related to other viruses have also been reported (Table 2), and their strategies are roughly the same. Specific DNAzymes were designed for specific cleavage of some conservative regions or the expression of some key genes in the virus genome. The results showed that these DNAzymes had good cleavage efficiency *in vitro*, effectively inhibiting the occurrence and spread of the virus.

**TABLE 2:** DNAzymes target viral targets genes.

Virus	mRNA target	DNAzyme	Result	References
HBV	X	Dz-237 and Dz-307	The content of X protein in liver-specific cell line HepG2 was significantly reduced	Goila and Banerjea (2001)
	s and e	DrzBS and DrzBC	The expression of HBV s or e genes in 2.2.15 cells was depressed dramatically	Wo et al. (2005)
	C	Drz-HBV-C-9	Drz-HBV-C-9 efficiently cleaved C gene mRNA at specific sites *in vitro.*	Hou et al. (2005)
	DR1 and polyadenylation signal regions	DR1Dz	DR1Dz significantly inhibited the expression of the HBV Luciferase fusion gene in Huh 7 cells, up to 48% of the control.	Asahina et al. (1998)
HCV	5´—NCR and core region	Dz2 and Dz4	Dz2 and Dz4 significantly inhibited the expression of the HCV-luciferase fusion gene in Huh7 cells.	Oketani et al. (1999)
	IRES	Dzs	Most Dzs effectively inhibit HCV IRES-mediated translation	Roy et al. (2008)
	NS5B	NDz	The mRNA and protein expression of NS5B were significantly reduced.	Kumar et al. (2009)
	NS3	-	Effectively reduced the expression of HCV NS3 and inhibited HCV RNA replication	Lee et al. (2010)
Influenza A	PB2	PB2Dz	PB2Dz inhibited influenza virus replication in cells.	Toyoda et al. (2000), Takahashi et al. (2000)
	M1	M1Dz	M1Dz cleaved M2 mRNA in a dose-dependent manner	Kumar et al. (2012)
	M2	Dz114	Dz114 inhibited M2 gene expression by up to 70%, the mutant-Dz against M2 gene showed no downregulation	Kumar et al. (2015)
Influenza B	BM2	Dz209	Significantly inhibited the expression of the BM2 gene in cells.	Kumar et al. (2013)
RSV	N	DZn1133	Effectively cleaved RSV RNA *in vitro*, inhibited transcription and expression of F virus genes, reduced RSV production, and protected RSV-infected Hep-2 cells from cytopathic effects.	Xie et al. (2006), Zhou et al. (2007)
SARS-CoV	5´—UTR	Dz-104	Effectively cleaved RNA substrates *in vitro* and inhibited the expression of 5´ UTR - eGFP fusion RNA in mammalian cells.	Wu et al. (2007)
JEV	3´—NCR	Dzs	Dzs inhibited JEV replication in cultured mouse cells of neuronal and macrophage origin	Appaiahgari and Vrati (2007)
human papillomavirus type 16	E6 and E7	Dz1023-434	Effectively inhibited the expression of HPV-16 E6/E7 mRNA	Reyes-Gutiérrez and Alvarez-Salas (2009)

DR1: Direct repeat 1.

5´- NCR: 5´—noncoding region.

IRES: internal ribosome entry site.

NS3: Nonstructural gene 3.

N: nucleocapsid.

5´—UTR: 5´- untranslated region.

RSV: respiratory syncytial virus.

JEV: japanese encephalitis virus.

3´—NCR: 3´—noncoding region.

### 3.6 DNAzymes targeted therapy for central nervous system disease

#### 3.6.1 Inhibition of HD protein

Huntington´s disease is an autosomal dominant genetic disease caused by the amplification of CAG repeat sequences in the Huntington´s (HTT) gene, resulting in the selective deletion of neurons in HD (Bañez-Coronel et al., 2012). Although the molecular events that lead to neuronal death are unclear, mutated HD proteins are likely to operate through a “functional acquisition” mechanism (Yen et al., 1999). Yen et al. (1999) tried to reduce the level of mutated HD protein by inhibiting the expression of HD mRNA. They designed a specific DNAzyme to cleave HD mRNA effectively and found that the DNAzyme not only cleft the target in a sequence-specific manner but also effectively reduced the content of HD protein expression in mammalian cells. Therefore, DNAzyme inhibiting the expression of HD protein may be an effective treatment for Huntington’s disease in the future.

#### 3.6.2 Inhibition of GAG chains

Scar tissue in the damaged spinal cord inhibits axonal growth, mainly related to the inhibitory molecule GAG chain. Therefore, inhibiting the formation of GAG chains is a key target for treating spinal cord injury. Since the formation of glycosylation of the GAG chain is catalyzed by xylosyltransferase 1 (XT-1), Grimpe et al. (Grimpe and Silver, 2004) designed a specific DNAzyme (DNAXTas) targeting XT-1 mRNA. The results showed that DNAXTas reduced the GAG chain in TGF-b stimulated Astrocyte transfected *in vitro* and improved the axon growth of Dorsal root ganglion neurons in injured rat spinal cord. Further research also showed that DNAXTas reduced the expression of GAG chains and core proteins of neural stems and short peptides in injured/transplanted spinal cord and inhibited scar tissue obstruction at the interface of the injured spinal cord, thereby restoring endogenous axon growth (Hurtado et al., 2008). To further explore the efficacy of DNAXTas *in vivo*, Oudega et al. (2012) administered DNAXTas intravenously and found that this systemic administration could lead to a significant increase in sensorimotor function and 1-hydroxytryptaminergic axons in the injured tail and would not cause toxic effects and exacerbate Neuropathic pain caused by contusion. A study also showed that DNAXTas treatment could significantly improve the growth of the corticospinal tract and had a long-term therapeutic effect, providing new insights into the regeneration process (Koenig et al., 2016). These findings confirm that DNAzymes may become a potential treatment strategy for repairing spinal cord injury.

In conclusion, DNAzymes can effectively inhibit the expression of target genes in these diseases both *in vivo* and *in vitro*. In addition, DNAzymes further verified the key role of some targets in disease. In the future, we will use DNAzymes as an important tool to study diseases. However, although many cell and animal disease trials have shown that DNAzymes have a good therapeutic efficacy, there has yet to be much preclinical research. Only a few DNAzymes have entered clinical trials mainly to treat cancer and chronic inflammatory diseases (Table 3). It is worth noting that Dz13 targeting c-jun was first tested in phase I clinical trials in patients with nodular basal cell carcinoma from 2010 to 2012, the results showed that Dz13 is safe, effective, and well tolerated (Cho et al., 2013). However, there has been no corresponding progress in subsequent clinical trials so far. Three different DNAzymes (SB010, SB011, and SB012) are being studied by Sterna Biologicals to treat asthma, atopic dermatitis, and ulcerative colitis. From the current experimental results, SB010, SB011, and SB012 containing hgd40 formulation components have shown good therapeutic effects. We expect more DNAzymes to enter clinical trials in the next clinical research.

**TABLE 3:** DNAzymes in clinical trials.

mRNA target	DNAzyme	Condition	Phase	Dosage regimen	References/Identifier
c-jun	Dz13	Nodular basal-cell carcinoma	Phase I completed	Three dose groups (10, 30, and 100 μg of Dz13) with three patients per group. Single intratumoral administration of 50 μL of Dz13 over 4 weeks	Cho et al. (2013)
LMP1	DZ1	Nasopharyngeal Carcinoma	Phases I/II completed	DZ1 in saline is administrated by intratumoral injection 2 hours prior to radiation therapy from week 1 to week 7 on Monday and Thursday. The dosage for each injection is 12 mg in 0.1 mL (200 μg per kilogram body weight.	Cao et al. (2014)
GATA—3	SB010	Asthma	Phases I/IIa completed	Inhale 10 mg of SB010 once a day for 28 days	Krug et al. (2015)
		chronic obstructive pulmonary disease	Phases IIa completed	Inhale 10 mg of SB010 once a day for 28 days	Greulich et al. (2018)
GATA—3	SB011	Atopic dermatitis	Phase I/IIa completed	Topical application of approximately 5 mg/cm^2^ (250 μL) per treatment area (50 cm^2^) twice daily on 14 consecutive days, one single last application at the site on Day 15 (29 treatments) Daily dosage: Approximately 10 mg hgd40 Total dosage: Approximately 145 mg hgd40	(NCT02079688)
GATA—3	SB012	Ulcerative colitis	Phases I/IIa completed	SB012 was provided in 30 mL PBS at a 7.5 mg/mL hgd40. The maximum daily dose will not exceed 225 mg. The treatment phase lasts 28 consecutive days.	(NCT02129439)

## 4 Delivery strategy of DNAzymes

Although DNAzymes significantly reduce the expression of related genes in the disease process, they are still interfered with by nuclease degradation, low cell uptake efficiency, and chemical weapons of natural DNA or RNA in practical applications. Therefore, improving the safety and effectiveness of DNAzymes *in vivo* is also a major challenge. Here, we introduce the methods based on chemical modification and the development of some delivery materials to effectively protect DNAzymes from the interference of other substances and increase cellular uptake.

### 4.1 Chemical modification method

**FIGURE 3:**
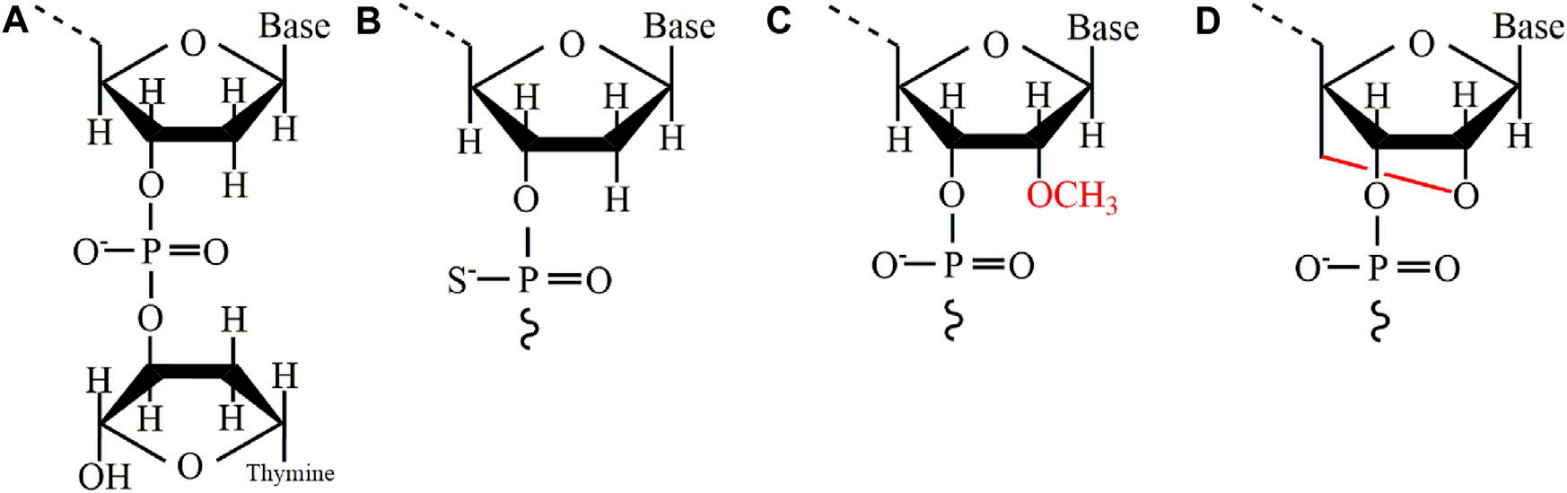
Some main modification methods for DNAzymes. **(A)** 3´-inversed dT **(B)** Phosphorothiate **(C)** 2´- O—methylation **(D)** Locked nucleic acid (LNA).

Chemical modification is a commonly used method to improve the catalytic activity and stability of DNAzymes. At present, some of the main modification methods include 3´—inversed dT, Phosphorothiate (PS), 2´—O—methylation, and Locked nucleic acid (LNA). Table 4 summarizes the advantages and disadvantages of each modification. From the current clinical application, incorporating 3´—inversed dT into the 3´ end of DNAzymes is the most widely used modification method (Figure 3A). This modification can effectively prevent the degradation of nucleic acid exonucleases, effectively extending the half-life of DNAzymes in human serum (Sioud and Leirdal, 2000). Compared with unmodified DNAzymes, DNAzymes with this modification can maintain functional integrity for a long time after exposure to serum, with almost no changes in kinetics (Dass et al., 2002). Another important and commonly used modification is PS (Figure 3B). This modification is used first to protect antisense oligonucleotide from the degradation of serum endonuclease and exonuclease and later to protect DNAzymes (Sioud and Leirdal, 2000; Alama et al., 1997). Secondly, as shown in Figure 3C, 2´—O—methylation is a natural modification widely used in antisense oligonucleotides and aptamers to protect DNAzymes from nuclease degradation and enhance the cutting efficiency of DNAzymes in cells (Schubert et al., 2003; Fokina et al., 2012). In addition, a more popular modification is LNA, a special double-strand stabilizer successfully used to modify siRNA (Elmén et al., 2005) and antisense oligo nucleotide (Shimo et al., 2014). As shown in Figure 3D, this modification has also become an attractive monomer for modifying DNAzymes to increase binding affinity, improving the stability and cutting efficiency of DNAzymes, and directly delivering cells (Fahmy and Khachigian, 2004; Vester et al., 2002).

**TABLE 4:** Advantages and disadvantages of each modification.

Chemical modification	Advantage	Disadvantage	References
3´—inversed dT	Resist the degradation of nucleic acid exonucleases, improve stability and catalytic rate	Slower product release rate, relatively short half-life	Dass et al. (2002); Fahmy and Khachigian. (2004); Chakravarthy et al. (2017)
Phosphorothiate (PS)	Improved stability, increases cellular uptake	Decrease substrate affinity, toxic side effects	Bhindi et al. (2007)
2´—O—methylation	Improved stability, increased oligonucleotide binding affinity	Reduced cleavage ability	Schubert et al. (2003); Robaldo et al. (2012)
Locked nucleic acid (LNA)	Improved stability and binding to highly structured RNAs	Affect catalytic activity and biological potency	Vester et al. (2002); Jadhav et al. (2009)

### 4.2 Delivery system

In recent years, with the development of nucleic acid therapy drugs, lipid nanoparticles, polymer nanoparticles, inorganic nanoparticles and other delivery materials have become increasingly popular. Therefore, many studies have reported that these materials can effectively deliver DNAzymes to the target *in vivo* and improve stability and cell uptake efficiency.

#### 4.2.1 Lipid nanoparticles

Among lipid nanoparticles, liposomes are the earliest to be used for delivering small-molecule drugs. The surface charge divides into liposomes cationic, neutral, or anionic liposomes. This liposome-based nanocarrier has been widely used in the research of nucleic acid therapy drugs, especially antisense oligonucleotides, and siRNA (Fan et al., 2021). Cationic liposomes are used most frequently in most disease models studied by DNAzymes *in vivo* and *in vitro*. With the progress of phase I clinical trials targeting the treatment of nodular basal cell carcinoma with Dz13, DOATP, and DOPE, cationic liposomes were delivered to Dz13 through local intratumoral injection and found safe, effective, and well tolerated. This preparation was also studied and evaluated during vein transplant stenosis in New Zealand white rabbits, as this process mainly causes smooth muscle cell proliferation (SMC). Dz13 targeting c-jun mRNA cleavage was transfected and found to reduce SMC proliferation and c-jun expression *in vitro* significantly. The formed lipid complex is also stable and allows sufficient venous absorption (Li et al., 2013).

With the continuous deepening of research, liposomes have gradually developed into lipid nanoparticles (LNPs) currently receiving attention mainly due to the recent successful delivery of siRNA and mRNA vaccines by LNPs in clinical applications (Suzuki and Ishihara, 2021; Chaudhary et al., 2021). Specifically, if the mRNA vaccine is not coated with LNP, its activity in the body may be lost in the delivery of mRNA vaccines. LNP mainly consists of four components: ionized cationic lipids, phospholipids, cholesterol, and polyethylene glycol lipids. Among them, ionized cationic lipids are crucial in helping mRNA achieve endosomal escape. Theoretically, DNAzymes are effectively bound by ionized cationic lipids, and this preparation seems to be used to deliver DNAzymes in the future. However, no relevant research has been reported so far, which may be because DNAzymes have not reached the same stage of development as other nucleic acid therapy drugs and are only based on the delivery of early liposomes.

#### 4.2.2 Polymer nanoparticles

Polymer nanoparticles have also received research and attention in the delivery of DNAzymes, mainly because polymer nanoparticles have good biodegradability and biocompatibility. For example, the common cationic polymer has many positive charges. In this case, DNAzymes are only regarded as a general nucleic acid to combine with the cationic polymer effectively.

In an early study, Khan et al. (2004) encapsulated DNAzymes in the copolymer of polylactic acid and polyglycolic acid (PLGA) microspheres by the method of double lotion deposition and studied the drug release characteristics *in vitro* after it was encapsulated in PLGA microspheres. They found that the release profiles of these DNAzymes were biphasic, first ushered in an explosive effect in the initial stage and then achieved continuous release in the second stage. This encapsulation´s attempt shows the delivery system´s potential to realize the continuous release and accumulation of DNAzymes. However, this study did not further test the content of DNAzymes initially embedded in the microspheres and the activity after release.

Polyamide amine dendrimers (PAMAM) are currently widely used polymer nanoparticles with high cellular characteristics and transfection efficiency due to their easy structure and surface modification (Tarach and Janaszewska, 2021). For example, the arginine-modified PAMAM dendrimer e-PAM-R had been used to deliver DNAzymes in cells, compared with the PAMAM-R series containing amide bonds as gene carriers, e-PAM-R G4 showed high transfection efficiency and cell viability in cells and effectively inhibited the expression of TEL/AML1 protein, this biodegradable and non-toxic e-PAM-R may be a useful carrier for subsequent delivery of DNAzymes (Nam et al., 2008). A recent study also developed phenylboronic acid functionalized polyamide (PP) for intracellular delivery of Dz13, and PP exhibited excellent transfection efficiency due to the sialic acid-dependent endocytosis pathway, which effectively inhibited the proliferation, migration and invasion of cancer cells (Yang et al., 2019). These studies indicate that PAMAM provides a potential delivery route for DNAzymes-targeted gene therapy in the future.

Various modified polypropylene imide dendrimers are also used to deliver DNAzymes. For example, by modifying PPI dendrimers with acetyl or ethylene glycol gallate (PEG) groups at the external primary amine and methyl iodine (MeI) or MeCl at the internal tertiary amine, multiple quaternary cation sites are generated. All modified PPI dendrimers exhibited low cytotoxicity and high cell uptake efficiency *in vitro*. The fourth-generation polypropylene imine dendritic polymer tested had a transfection efficiency of nearly 80% and had been confirmed *in vivo* through intravenous injection in nude mice (Tack et al., 2006).

Chitosan is a naturally occurring cationic polymer with good biocompatibility and the ability to bind to DNA through electrostatic interactions. Some researchers have synthesized nanoparticles formed from chitosan, which are used for packaging Dz13 for delivery to osteosarcoma cells, and found that Dz13 not only effectively inhibited the growth of osteosarcoma but also stabilized in serum for 1 week and at room temperature for 1 month (Dass et al., 2008a). Recently, different hydrogel formulations based on chitosan had also been developed and tested for their permeability and the protective effect of DNAzymes degradation, the developed formulations showed good antibacterial activity even without the addition of preservatives, and the recovery of full layer pig skin samples showed that the preparation provides good skin permeability (Eicher et al., 2019).

Other polymer nanoparticles, such as β—Cyclodextrin polymer nanoparticles, were also used to deliver DNAzymes targeting oncogene c—Myc in human breast cancer cell lines. The results showed that the complex effectively inhibited the expression of the c—Myc gene and enhanced the synergistic effect with the adriamycin, which was superior to free drugs (Farrokhi et al., 2018). In another study, to further restore the drug sensitivity of breast cancer cell lines to doxorubicin, Zokaei et al. (2019) coupled cyclodextrin particles with chitosan to deliver DNAzymes targeting MDR1 mRNA. MDR1 is responsible for encoding the expression of drug resistance genes and plays an important role in the acquired drug resistance of anti-cancer drugs. After a series of tests, it was found that the interaction between this complex and DNAzymes significantly decreased the expression of MDR1 and increased drug sensitivity. In addition, it also increased the accumulation of DNAzymes in the treated cells.

#### 4.2.3 Inorganic nanoparticles

Among inorganic nanoparticles, gold nanoparticles (AuNPs) are a popular delivery material for DNAzymes, mainly due to the inherent optical properties of AuNPs and simple surface chemical modification with various ligands. DNAzymes could be coupled with gold nanoparticles to achieve the key restriction of entering cells. This coupling compound enhances the stability of DNAzymes and their ability to enter mammalian cells and effectively regulates their catalytic activity in cells (Yehl et al., 2012). To verify the activity of this coupling compound *in vivo*, Somasuntharam et al. (2016) conducted experiments in mice by local injection. The results showed that this coupling compound effectively knocked out the expression of 50% tumor necrosis factor, which is also the first study that gold nanoparticles are used to deliver DNAzymes and regulate genes *in vivo*. These studies may provide new opportunities for DNAzymes in subsequent therapeutic applications.

Other inorganic nanoparticles have been studied to deliver DNAzymes, including iron oxide, titanium oxide, and arginine-modified hydroxyapatite nanoparticles. For example, Ryoo et al. (2012) coupled DNAzymes, cell-penetrating peptide (MPAP) and dextran-coated magnetic iron oxide nanoparticles. The results showed that the nanocomposite would not cause adverse immune reactions in Huh-7 cells *in vitro* and had a higher knockout effect than the “naked” DNAzymes transfected with Lipofectamine (TM) 2000. When mice are injected through the tail vein, nanocomposites can accumulate in the liver (Ryoo et al., 2012). In addition to iron oxide rice particles, arginine-modified nano-hydroxyapatite particles (Arg-nHAP) had been confirmed to be used to deliver Dz1 in the CNE1-LMP1 xenotransplantation model of mice. Arg-nHAP and Dz1 had high transfection efficiency under *in vitro* conditions, and the use of specific inhibitors confirmed that the cell uptake of Arg-nHAP and Dz1 complex was mediated by energy-dependent endocytosis, The confocal microscope observed effective intracellular delivery and nuclear localization of the complex and also significantly downregulated the expression of LMP1 in nasopharyngeal carcinoma cells and inhibited tumor growth in mice (Chen et al., 2013). In addition, Levina et al. (Levina et al., 2014) coupled DNAzymes with titanium oxide nanoparticles. Although the activity of DNAzymes in the complex was slower than that of free DNAzymes, it had the same site specificity and cutting efficiency. More importantly, the complex could penetrate cells well without transfectants.

#### 4.2.4 Other delivery materials

In addition to the delivery mentioned above carriers, some other delivery carriers are also gradually gaining popularity. For example, metal-organic frameworks (MOFs) have become promising drug carriers and do not require the study of components that decompose *in vivo*. Recently, Wang et al. (Wang et al., 2019) reported a self-sufficient MOFs-based chloride protein e6 modified DNAzymes (Ce6 DNAzymes) treatment nanosystem for combining gene and photodynamic therapy. In this nanosystem, ZIF-8 nanoparticles were decomposed with the release of DNAzymes and Zn under PH response, and the released Zn further provides a cofactor for DNAzymes *in vivo*. In addition, the reactive oxygen species generated by the auxiliary photosensitizer Ce provide fluorescence signals for image-guided gene therapy and photodynamic therapy. This strategy makes up for the lack of cofactor of DNAzymes *in vivo* and realizes multiple therapeutic approaches. Similar studies have also reported that this MOF-based chemical genomic therapy was used to coat MnO_2_ nanosheets to provide doxorubicin and self-activated DNAzymes for cancer (Nie et al., 2020). The use of MOFs for delivering DNAzymes is receiving extensive research and attention. In addition to ensuring efficient gene therapy by DNAzymes, it is also used for biological analysis and biological imaging of DNAzymes *in vitro* and *in vivo* (Wu et al., 2020).

With the development of DNA nanotechnology, various DNA nanostructures with actual sizes and shapes have been reported as potential carriers for DNAzymes delivery *in vivo*. Tetrahedral DNA nanostructures have demonstrated excellent biocompatibility, stability, and high cell permeability, Meng et al. (2019) directly added the targeted cleavage c-jun Dz13 sequence to the 5—terminus of single-stranded DNA to form a modified tetrahedral DNA nanostructure (TDN-Dz13) for delivering Dz13 into cells. They found that TDN-Dz13 had high cell uptake efficiency and effectively inhibited c-jun expression within cells. This study may have a good attraction for the future delivery of DNAzymes.

Some other DNA nanomaterials also have great advantages, such as a bionic self-catabolic DNA nanocapsule, which realizes the specific cascade activation of DNAzymes in cancer cells (Wang et al., 2021). Secondly, a dynamic DNA nano sponge realized tumor-targeted delivery and gene regulation of DNAzymes and showed good biocompatibility and stability (Luo et al., 2022). The discovery of these delivery vectors will probably promote the clinical application of DNAzymes and other nucleic acid therapy drugs as gene regulation tools.

Overall, these nanomaterials have significant application potential for delivering DNAzymes *in vivo*. In contrast, liposomes have efficient cell uptake efficiency, polymer nanoparticles have good biocompatibility and biodegradability, and inorganic nanomaterials have good biocompatibility, large surface area, and easy surface functionalization. One key advantage of DNA nanocarriers compared to other delivery carriers is their unique programmability and ability to control drug distribution space, indicating that DNA nanocarriers have good application prospects (Charoenphol and Bermudez, 2014). However, these delivery carriers also have some limitations in practical applications, such as the high toxicity of liposomes and polymer nanoparticles to cells, low encapsulation efficiency, poor storage stability, and slow internal body escape of inorganic nanoparticles (Labatut and Mattheolabakis, 2018). DNA nanocarriers face many challenges, including size, shape, surface chemistry, and *in vivo* parameters that require systematic research (Kim et al., 2021). In addition, most of the delivered materials did not undergo corresponding *in vivo* experiments, and further observation of the effect *in vivo* is needed. In contrast, lipid nanoparticles are the most popular type and have good therapeutic effects in clinical applications. These delivery materials will be optimized and improved to make DNAzymes more likely to be applied in clinical practice in the near future.

## 5 Summary and outlook

Overall, we reviewed the progress of DNAzymes as a therapeutic agent in the past 20 years. Based on existing research results, DNAzymes have great potential for down-regulating the expression of related pathogenic genes. For example, Dz13, ED5 and hgd40 are potential candidates for clinical treatment targeting cancer, cardiovascular disease, and inflammatory disease. With the continuous discovery of more and more gene expressions that lead to disease states, DNAzymes target these new target genes to achieve therapeutic effects on diseases. However, DNAzymes still face some challenges in practical applications, such as improving stability and catalytic activity *in vivo*, and ensuring sustained and efficient drug delivery and targeting. However, these will be solved with the ease of modification of DNAzymes and the development of some delivery materials, such as metal-organic frameworks, DNA nanocarriers, and lipid nanocarriers, which have great potential in delivering DNAzymes. we need to know how to put DNAzymes into clinical applications. With the continuous deepening of research, several DNAzymes have entered clinical trials, and the experimental results obtained are well tolerated, safe, and effective. In the future, we hope that clinical trials of DNAzymes can be smoothly conducted and put into clinical applications, and we also look forward to more mature nucleic acid-based therapies. DNAzymes can emerge as a useful research tool in biomedicine, helping humans effectively treat various diseases.
